# Citric Acid Inhibits Cd Absorption and Transportation by Improving the Antagonism of Essential Elements in Rice Organs

**DOI:** 10.3390/toxics12060431

**Published:** 2024-06-14

**Authors:** Kexin Chen, Bozhen Yu, Weijie Xue, Yuebing Sun, Changbo Zhang, Xusheng Gao, Xiaojia Zhou, Yun Deng, Jiarun Yang, Boqian Zhang

**Affiliations:** 1 Key Laboratory of Original Agro-Environmental Pollution Prevention and Control, Agro-Environmental Protection Institute, Ministry of Agriculture and Rural Affairs, Tianjin 300191, China; ckx_1_1@163.com (K.C.); 18604519599@163.com (B.Y.); sunyuebing@caas.cn (Y.S.); zhangchangbo@caas.cn (C.Z.); 13001303720@163.com (X.G.); zxj103698@163.com (X.Z.); 2 School of Environment and Ecology, Jiangnan University, Wuxi 214122, China; dengyun@jiangnan.edu.cn; 3 College of Water Conservancy Engineering, Tianjin Agricultural University, Tianjin 300392, China; 18987184564@163.com (J.Y.); 15702675200@163.com (B.Z.)

**Keywords:** Cd, rice, citric acid, foliar application, essential element

## Abstract

Excessive cadmium (Cd) in rice is a global environmental problem. Therefore, reducing Cd content in rice is of great significance for ensuring food security and human health. A field experiment was conducted to study the effects of foliar application of citric acid (CA) on Cd absorption and transportation in rice under high Cd-contaminated soils (2.04 mg·kg^−1^). This study revealed that there was a negative correlation between Cd content in vegetative organs and CA content, and that foliar spraying of CA (1 mM and 5 mM) significantly increased CA content and reduced Cd content in vegetative organs. The Cd reduction effect of 5 mM CA was better than that of 1 mM, and 5 mM CA reduced Cd content in grains and spikes by 52% and 37%, respectively. CA significantly increased Mn content in vegetative organs and increased Ca/Mn ratios in spikes, flag leaves, and roots. CA significantly reduced soluble Cd content in vegetative organs and promoted the transformation of Cd into insoluble Cd, thus inhibiting the transport of Cd from vegetative organs to grains. The foliar field application of 1 mM and 5 mM CA could inhibit Cd absorption and transportation by reducing Cd bioactivity and increasing the antagonistic of essential elements in rice vegetative organs. These results provide technical support and a theoretical basis for solving the problem of excessive Cd in rice.

## 1. Introduction

Due to industrial development and human activities, soil cadmium (Cd) pollution has become increasingly severe (reaching almost 7.75% on Chinese farmland) [1]. As the awareness of food safety grows, preventing the accumulation of Cd in cereal crops and its transmission through the food chain has become an urgent issue [2]. As one of the primary cereal crops, rice is at an increased risk of Cd exposure in Cd-contaminated soil. The excessive accumulation of Cd in rice not only affects the normal growth and development of rice quality and yield but also poses a threat to human health through the food chain, such as causing itai-itai disease and cancer [3]. Therefore, controlling rice Cd pollution has become a hot topic in recent research. With a deeper understanding of Cd absorption and transport mechanisms in rice, various approaches are widely applied to reduce rice Cd content, including soil improvement, bioremediation techniques, and molecular biology methods [4,5]. Among them, the foliar application of conditioning agents is an economically effective mean to inhibit Cd absorption and transport [6,7].

Citric acid (CA) is a crucial substance in many core biological metabolic reactions. As a small-molecule organic acid, it has been confirmed to enhance plant tolerance to heavy metals, maintain cellular metabolism, and improve resistance to heavy metal stress [8,9]. Research has shown that citric acid-modified biochar can reduce the bioavailability of Cd in soil [10]. Citric acid could regulate the balance of anions and cations in rice by mediating the balance of cations and anions within plants and subsequently affecting the transport of mineral elements within plant tissues [11,12]. Exposed to 50 μM Cr stress, the CA content significantly increased in rice. Under Al stress, the roots of rice could enhance CA content to counter heavy metal toxicity [13,14]. Furthermore, the external application of CA has been found to enhance antioxidant capacity in pea seedlings, thereby increasing their tolerance to Cu [15]. Additionally, CA upregulated the iron transport protein (*OsIRT1*), competed with Cd-related transport proteins, and thus mitigated Cd stress in rice [16,17]. However, research has also indicated that CA enhanced the hyperaccumulation of heavy metal ions in plants [18,19]. Our earlier studies revealed that Cd stress induced rice roots and grains to secrete more CA to mitigate Cd toxicity. Different organs of rice could regulate the transport of Cd into grains. When the stress of Cd was severe, the intrinsic protective mechanisms of these organs were gradually weakened in resisting Cd stress [20]. It remains unclear whether the foliar application of CA could further enhance the blocking effect of Cd in various organs of rice to reduce the accumulation of Cd in rice under high Cd stress conditions.

The transport and distribution of mineral elements after organic acid application is also one way for plants to resist Cd stress. As basic crop nutrients, mineral elements (K, Ca, Mg, Mn, Fe, Zn, etc.) could affect the ability to absorb and accumulate Cd in plants [21]. For example, Mn, Fe, and Cd were absorbed and transported in the same pathway, thus the reduction in Mn and Fe content in rice promoted Cd accumulation in shoots [22]. The deficiency of Ca and Mg enhanced the toxicity of Cd in roots and shoots of rice seedlings by decreasing the antioxidant capacity of rice. The transport of Cu caused oxidative stress, triggering the signaling pathways in *Arabidopsis thaliana*, thus enhancing Cd resistance [23,24,25]. It has also been found that Cd could enhance K translocation from hyacinth roots to other tissues and reduce the uptake of Zn [26], while exogenous CA also influenced the absorption capacity of heavy metals by regulating plant mineral element content. There are no specific transport channels for Cd in plants, it mainly relies on the transport channels of other mineral elements, such as Mn transporters, so there is a competitive effect between Cd and Mn. It has now been shown that, under Cd stress, CA could modulate Mn transport to inhibit the uptake of Cd [27]. Treatment with 50 μM Mn increased the content of CA by 55.3% in the plant, thereby reducing Cd-induced oxidative damage [28]. However, the effect of the foliar application of CA on the uptake and transport of mineral elements in various organs of rice is still uncertain.

The period from panicle initiation to flowering is the main phase for rice to absorb chemical elements [29]. The flowering stage is the key period for Cd to be reactivated, redistributed, and transported to the grain within different organs of the rice, ultimately directing it toward the grains [20,30]. Spraying foliar regulators during flowering could inhibit the remigration of Cd in nutrient organs, avoiding excessive transport of Cd from vegetative organs to grains. Spraying 5 mM nano-silicon (Si) 1–2 times from the tillering to the grouting stage of rice could reduce Cd content by 31.6% and 36.1% in rice and rachises, respectively [31]. It has been found that the flowering stage is also an important period to increase grain yield and reduce the influence of Cd [32,33,34]. In addition, citric acid can also chelate with Cd, reducing Cd activity in vegetative organs and thus avoiding Cd transportation to rice grains.

Therefore, field experiments were conducted to explore the effects of foliar spraying of CA on the absorption and transport of Cd and essential elements in different organs of rice under high Cd stress. By analyzing the correlation between CA and Cd in different organs of rice and the differences in Cd transfer factors, the mechanism of foliar spraying of CA to reduce the content of Cd in rice was explored, which may provide a practical basis for subsequent research to develop novel foliar conditioning agents for the control of Cd pollution in rice.

## 2. Materials and Methods

### 2.1. Plant Materials and Experimental Site

A high-yielding and high-quality late modern indica rice “Huazhan” (widely cultivated in Hunan Province) was used in this study. The cultivation took place in red soil paddy fields located in the northeastern part of Hunan Province (N: 28°49′, E: 112°50′). This region experiences relatively high Cd contamination in rice fields, with a Cd content of 2.04 mg·kg^−1^ and similar fundamental chemical properties. Specifically, topsoil pH was 5.48; CEC was 20.2 cmol·kg^−1^; organic matter content was 43.85 g·kg^−1^; K, Ca, Mg, and Fe were 10.1, 21.2, 30.8, and 32.9 g·kg^−1^, respectively; Mn and Zn were 286.4 and 125.8 mg·kg^−1^.

### 2.2. Experimental Design and Treatments

The experiment included a control group (CK, treated with surfactant) and two treatment groups (S1: treated with 1 mM CA + surfactant; S2: treated with 5 mM CA + surfactant). We employed a completely randomized block design, with three replicates for each group. The plot dimensions were 2.5 m (length) × 4 m (width), resulting in an area of 10 m^2^. Rice was sown in June 2018 and transplanted to the paddy fields in late July. To avoid any impact on the soil, during the rice flowering stage (late September), different concentrations of citric acid were uniformly sprayed on rice leaves. Field management practices were consistent with those used in high-yield rice fields in Hunan.

### 2.3. Sample Processed and Collection

During the rice maturation stage, fresh rice samples were collected from each paddy field using a five-point sampling method. After washing with deionized water, the samples were divided into the following components: grains, panicles (including the sheath), flag leaves, spikes, necks, remaining shoot parts, and roots. Each component was further split into two portions.

One portion was dried at 105 °C in an oven for 30 min, followed by drying at 70 °C to constant weight. The dried samples were ground through a 20-mesh nylon sieve and used for Cd and essential element analysis after digestion. The other portion was air-dried naturally in a cool ventilated area and used to determine different forms of Cd content in the plant organs.

### 2.4. Sample Parameter Determination

#### 2.4.1. Cd and CA Content Determination

Approximately 0.2500 g of dried rice samples from different plant parts was weighed into digestion tubes. Nitric acid (65.0–68.0 wt%, 8 mL, analytical reagent) was added, and the samples were soaked for 12 h. Subsequently, the samples were digested in an electric digestion instrument at 80 °C for 1.5 h, followed by 120 °C for 1.5 h, and then heated to 150 °C for 3 h. After digestion, the samples were heated to 160–180 °C to drive off excess acid (leaving 1 mL of digestion solution in the tube). The solution was cooled, diluted to 50 mL in a volumetric flask with distilled water, filtered, and analyzed using inductively coupled plasma mass spectrometry (ICP-MS, Agilent 7500a, Santa Clara, CA, USA).

For CA content determination, a plant citric acid assay kit (Solaibao, Beijing, China) was used for extraction and pretreatment. Absorbance was measured using a UV spectrophotometer, and the CA content was calculated based on a standard curve.

#### 2.4.2. Extraction of Cd Speciation

We weighed 0.3 g of plant samples and added 30 mL of 80% ethanol (sample-to-extractant ratio *w*:*v* = 1:100). The mixture was homogenized and shaken at 25 °C for 22 h. Then, we centrifuged it at 5000× *g* for 10 min, and the supernatant was collected in a clean container. We repeated this process twice and combined the supernatants from the three centrifugations. Next, we sequentially extracted the samples using different extractants. For each extractant, we evaporated the collected solution and the sample residue was dried on an electroplated plate at 70 °C to constant weight for subsequent analysis. The extraction and quantification of Cd chemical forms were adjusted based on previous methods [35,36]. The different extractants used were as follows: (1) 80% ethanol extraction (FE-Cd): extracts inorganic Cd; (2) deionized water extraction (FW-Cd): extracts water-soluble Cd. The two collected supernatants were combined to obtain soluble Cd, and the residual part was collected to obtain insoluble Cd.

#### 2.4.3. Determination of Mineral Element Content

An inductively coupled plasma mass spectrometer (ICP-MS, Agilent 7500a, Santa Clara, CA, USA) was used to measure the content of K, Mg, Ca, Fe, Mn, and Zn. The working conditions for ICP-MS were as follows: radiofrequency power of 1350W, plasma flow rate of 15.0 L·min^−1^, carrier gas flow rate of 1.12 L·min^−1^, and peristaltic pump speed of 6.0 rmp. A mixed standard solution was prepared (Agilent, Part # 5183-4688), with concentrations of 1000 μg·mL^−1^ for Ca, Fe, and K, and 10 μg·mL^−1^ for Mn and Zn. For the preparation of on-machine standard solutions, the concentration ranges were as follows: K, Ca, and Fe ranged from 0 to 40,000 μg·L^−1^, while Mn and Zn ranged from 0 to 400 μg·L^−1^. Standard curves were generated for each element.

#### 2.4.4. Transfer Factor Calculation

The transfer factor (TF) was used to analyze the transfer ability of soluble Cd between different rice organs. The calculation followed a method used by previous researchers [37]. The specific formula is as follows:

(1) $${\mathrm{TF}}_{A/B}\;=\;\frac{T_{A}}{S_{B}}$$

where TF_A/B_ stands for the transfer factor of soluble Cd between adjacent organs, T_A_ represents total Cd content in adjacent upper organs (mg/kg), and S_B_ stands for soluble Cd content in adjacent lower organs (mg/kg).

### 2.5. Data Analysis

The experimental data represent the average of three replicates. Significance analysis was performed using IBM SPSS Statistics 26, employing ANOVA and LSD methods to compare differences between different treatments (*p* < 0.05). Microsoft Excel 2021 and Origin 2022 were used for table and chart plotting.

## 3. Results

### 3.1. Effects of Leaf Spraying with CA on CA and Cd in Rice Organs

#### 3.1.1. CA and Cd Content and Correlation

**Figure 1:**
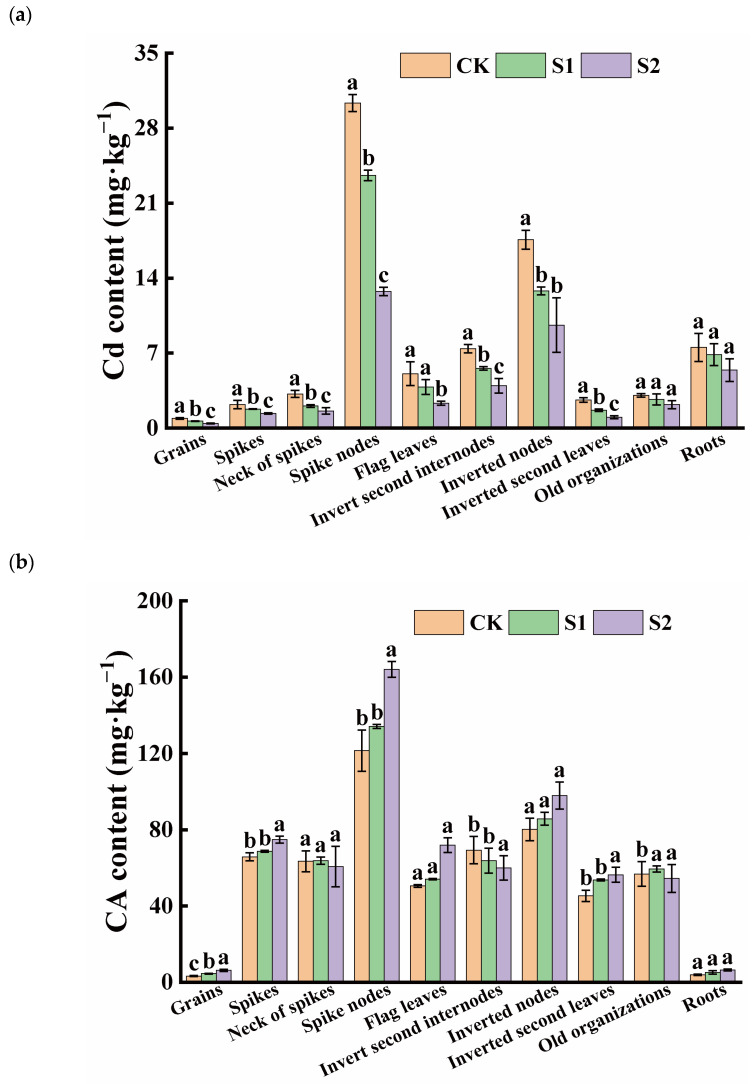
Content of Cd (**a**) and CA (**b**) in different tissues of rice. Different letters indicate significant difference (*p* < 0.05) among treatments in the same organ according to Duncan’s test.

The ability of Cd accumulation in rice organs varied significantly. The Cd content in rice roots was 7.53 mg·kg^−1^ under only Cd-contaminated treatment, while, among the shoots, the average Cd concentration was found in the spike nodes, reaching 30.33 mg·kg^−1^. The inverted nodes had a Cd content as high as 17.59 mg·kg^−1^, which was 4.03 and 2.34 times higher than in the roots, respectively. However, the Cd content in the rice grains was relatively low, approximately 0.90 mg·kg^−1^, exceeding both China’s grain safety standard (0.25 mg·kg^−1^) and the international grain safety standard (0.4 mg·kg^−1^). During the rice flowering stage, the foliar application of CA significantly increased the CA content in grains and vegetative organs, while reducing the Cd content in rice grains and their associated organs. The effect of spraying 5 mM CA was superior to that of 1 mM CA. Specifically, 1 mM and 5 mM CA significantly reduced Cd content in grains by 27.54% and 52.28%, respectively. Additionally, 5 mM CA significantly decreased Cd content in the necks of spikes, spike nodes, flag leaves, inverted second internodes, inverted nodes, and the inverted second leaves (Figure 1a). The lowest CA content was observed in rice grains and roots, while the CA levels in spike nodes were approximately 37.22 and 30.67 times higher than those in grains and roots, respectively. Furthermore, high concentrations of CA significantly increased the CA content in the spike nodes, flag leaves, inverted nodes, inverted second leaves, and roots, with no change in the necks of spikes, inverted second internodes, and old organizations (Figure 1b).

The Cd content of rice grains was positively correlated with the Cd content of shoot organs but not significantly correlated with the Cd content of the root system (Table 1). Except for rachis Cd content and old tissue Cd content, Cd content was positively correlated with shoot organs of rice. Citric acid content was significantly correlated among all rice organs except spike necks, binodal internodes, and old tissues. Cd and citric acid contents in seeds, rachis, nodes, flag leaves, inverted nodes, inverted leaves, and roots were negatively correlated, with the highest correlation coefficients of −0.92 and −0.94 between Cd and citric acid contents in seeds and nodes.

#### 3.1.2. Impacts of Foliar Application of CA on the Speciation of Cd in Various Organs of Rice

**Figure 2:**
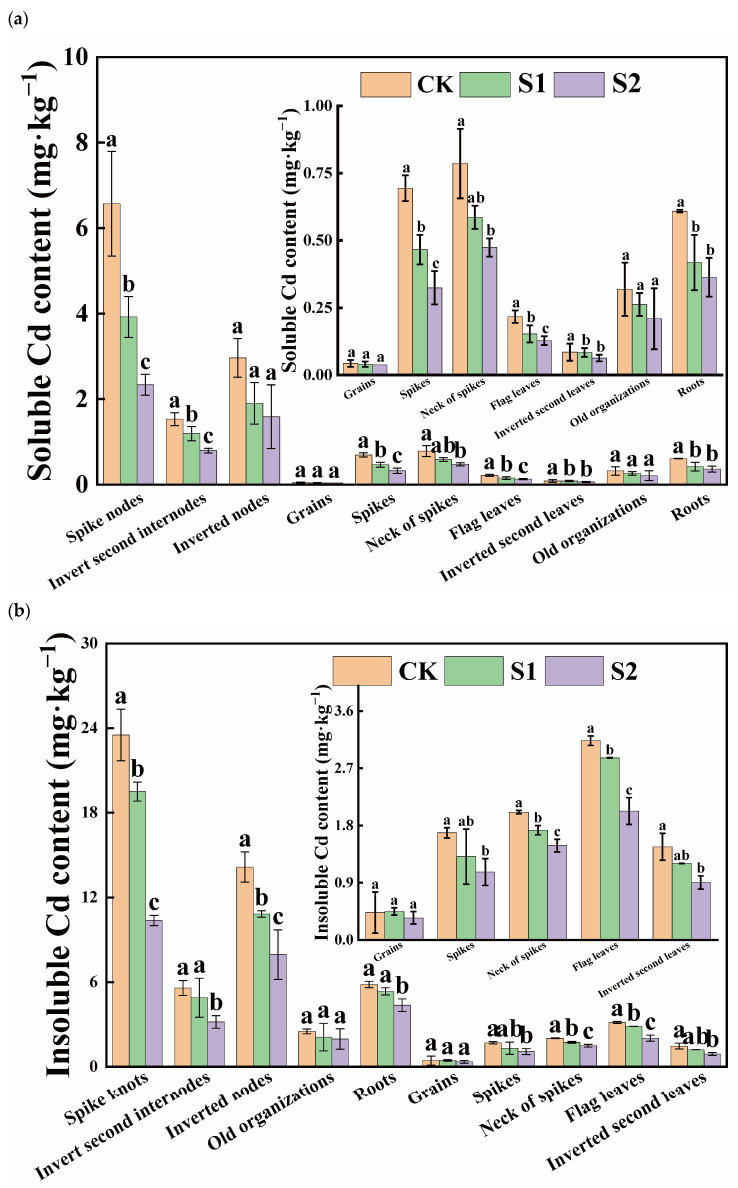
Content of soluble Cd (**a**) and insoluble Cd (**b**) in different tissues of rice. Different letters indicate significant difference (*p* < 0.05) among treatments in the same organ according to Duncan’s test.

Under Cd stress conditions, the insoluble form of Cd was the predominant speciation in various rice organs, and its Cd content far exceeded that of the soluble form. Notably, there was a significant difference in insoluble Cd content versus soluble Cd content in flag leaves, inverted second leaves, and roots, with ratios of approximately 14.23, 17.27, and 9.58, respectively. During the rice flowering stage, the foliar application of CA significantly reduced the soluble Cd content in organs, other than the inverted second leaves and old organizations. Moreover, the reduction effect of 5 mM CA was greater than that of 1 mM CA (Figure 2a). Compared with CK, 5 mM CA led to the following percentage decreases in soluble Cd content in various organs: spikes (53.32%), necks of spikes (39.75%), flag leaves (40.90%), spike nodes (64.44%), inverted second internodes (48.05%), inverted nodes (46.44%), and roots (40.36%), with substantial reductions observed in the neck and panicle nodes. Additionally, when compared with CK, 5 mM CA results in spikes, necks of spikes, flag leaves, inverted second leaves, spike knots, inverted second internodes, inverted nodes, and roots decreased by 36.64%, 26.27%, 35.32%, 38.29%, 55.95%, 43.09%, and 25.21% in insoluble Cd content, with significant reductions primarily observed in spike knots (Figure 2b). Overall, the inhibitory effect of 5 mM CA on soluble Cd content surpassed its effect on insoluble Cd content.

#### 3.1.3. Effects of CA on Cd Transport between Adjacent Organs in Rice

**Figure 3:**
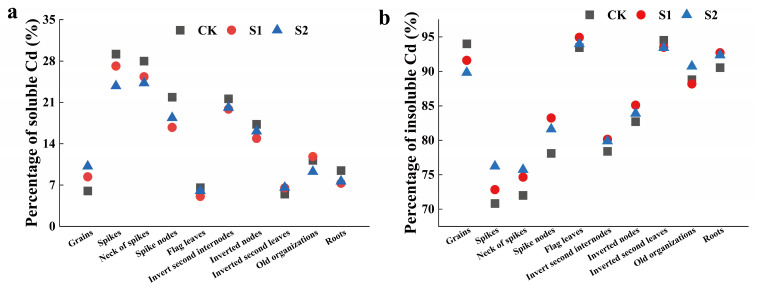
Percentage of soluble Cd (**a**) and insoluble Cd (**b**) in different tissues of rice.

The foliar application of CA could regulate the distribution ratios of soluble Cd and insoluble Cd in rice organs during the rice flowering stage. The regulatory effect of 5 mM CA surpassed that of 1 mM CA (Figure 3). Specifically, 5 mM CA significantly reduced the distribution ratios of soluble Cd in the spikes, necks of spikes, spike nodes, and roots by 18.52%, 13.33%, 16.02%, and 19.26%, respectively (Figure 3a). Conversely, 5 mM CA increased the distribution ratios of insoluble Cd in the spikes, necks of spikes, spike nodes, and roots by 7.63%, 5.18%, 4.49%, and 2.01%, respectively (Figure 3b).

Our experiments found that the transport capacity of soluble Cd to the inverted nodes was relatively strong, with flag leaves exhibiting an intermediate transport capacity to spike nodes. The transport capacity of soluble Cd from the spikes to the grains was weaker, while spike nodes exhibited the weakest transport capacity from soluble Cd to the spikes (Table 2). The spikes, spike nodes, flag leaves, and inverted second leaves played critical roles in the process of Cd transport toward the grains. Both 1 mM and 5 mM CA significantly inhibited the transfer of soluble Cd from the inverted second leaves to the inverted nodes, and 5 mM CA further suppressed the transport of soluble Cd from the flag leaves to the spike nodes. The foliar application of CA did not significantly affect the ability of other organs to transport soluble Cd to adjacent upper organs.

### 3.2. Essential Mineral Element in Various Organs of Rice

#### 3.2.1. Effects of Foliar Application of CA on Essential Mineral Element Content in Various Organs of Rice

Rice requires abundant essential mineral elements, including K, Ca, and Mg, and trace elements such as Mn, Fe, and Zn. The distribution of mineral elements varies significantly across different rice organs. During the rice flowering stage, the foliar application of CA had the most pronounced impact on the element content in grains, with the effect of 5 mM CA exceeding that of 1 mM CA (Table 3). Among the shoots, spike nodes consistently exhibited the highest element content, while grains had the lowest. Notably, the Fe in roots far exceeded that in other organs. Compared with CK, 5 mM CA significantly increased K, Mg, and Mn content in grains by 46.40%, 82.40%, and 18.93%, respectively, while reducing Ca and Zn content by 23.19% and 14.13%, respectively. Additionally, 5 mM CA significantly enhanced K in the spikes by 17.43% and Fe by 1.87 times. It also increased Mn content in spike knots by 23.99% but decreased Zn content by 17.03%. Furthermore, 5 mM CA led to a substantial increase in Mn content in the roots by 50.37%.

#### 3.2.2. Effects of Foliar Application of CA on the Speciation of Mn in Rice Spike Knots and Roots

**Figure 4:**
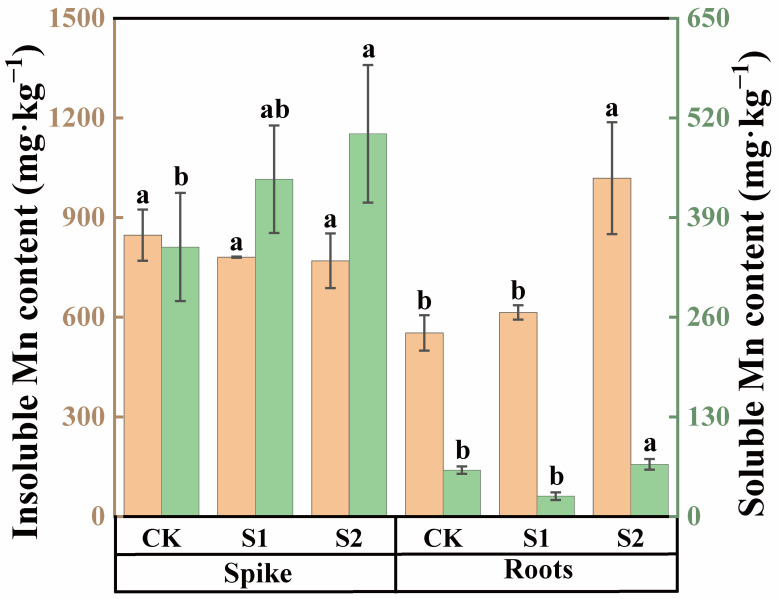
Content of soluble and insoluble Mn in spikes and roots of rice treated with different concentrations of CA. Data represent the means of three repetitions ± standard deviation and lowercase letters represent significant differences between different CA treatments (*p* < 0.05).

In both rice spike knots and roots, the content of insoluble Mn was higher than that of soluble Mn (Figure 4). Under Cd stress conditions, the insoluble Mn content in spike nodes was approximately 2.41 times higher compared with soluble Mn, while, in roots, the insoluble Mn content was 7.82 times higher than soluble Mn. The foliar application of CA could modulate the speciation of Mn within these organs during the rice flowering stage. An amount of 1 mM CA had no significant impact on the soluble Mn content in rice roots and spike nodes, as shown in Figure 4. However, compared with CK, 5 mM CA significantly increased the soluble Mn content in roots by 27.18% and in spike nodes by 42.06%. Neither 1 mM nor 5 mM CA significantly affected the insoluble Mn content in rice spike nodes. However, 5 mM CA substantially enhanced the insoluble Mn content in roots, with an impressive increase of 84.48%.

### 3.3. Effects of Foliar Application of CA on Ion Balance in Key Organs of Rice

#### 3.3.1. Effects of Foliar Application of CA on Mn:Cd Ratios in Different Organs

**Figure 5:**
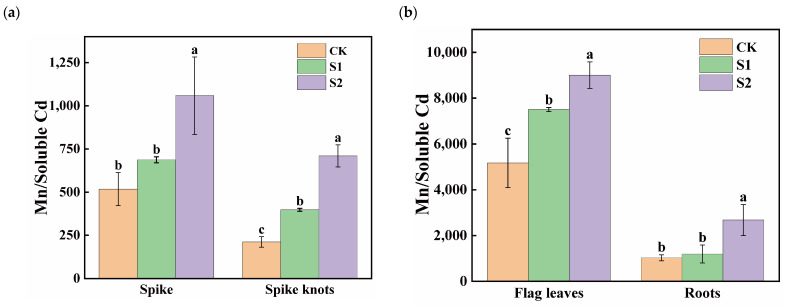
Effect of different concentrations of CA on Mn/soluble Cd in spikes and spike nodes (**a**) and flag leaves and roots (**b**) of rice. Data represent the means of three repetitions ± standard deviation and lowercase letters represent significant differences between different CA treatments (*p* < 0.05).

The ratios of Mn content to soluble Cd content in flag leaves was significantly higher than in spikes, spike nodes, and roots, with the smallest ratios observed in spike nodes (Figure 5). During the rice flowering stage, the foliar application of 1 mM CA significantly increased the Mn:Cd ratios in spike nodes by 87.68%. Moreover, 5 mM CA led to substantial ratio increases of 104.62% and 235.08% in both spikes and spike nodes (Figure 5a). Additionally, 1 mM CA significantly enhanced the ratios in flag leaves by 45.12%, while 5 mM CA significantly increased the Mn:Cd ratios in different organs in both flag leaves and roots; the increases were 74.20% and 160.12%, respectively (Figure 5b).

#### 3.3.2. Effects of Foliar Application of CA on the Ratio (Ca:Mn) to Soluble Cd Ratio in Different Organs

**Figure 6:**
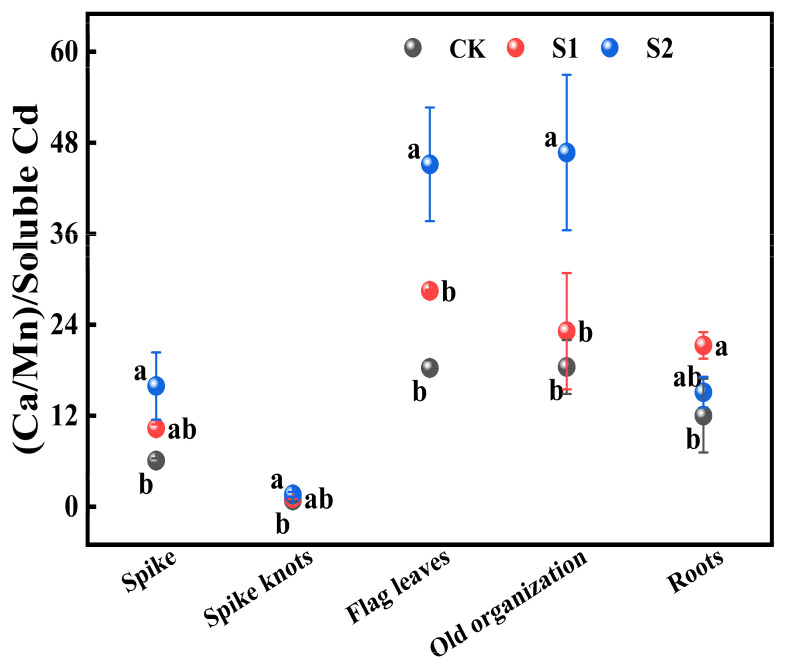
Impact of different concentrations of CA on (Ca:Mn)/soluble Cd in different key organs of rice. Data represent the means of three repetitions ± standard deviation and lowercase letters represent significant differences between different CA treatments (*p* < 0.05).

The ratio of (Ca:Mn) to soluble Cd in various rice organs from flag leaves, old organizations, roots to spike nodes showed a gradually decreasing trend. During the rice flowering stage, the foliar application of 1 mM CA significantly increased this ratio in all organs by 25.63% to 77.04% compared with CK. Additionally, 5 mM CA led to substantial increases in the ratio of (Ca:Mn) to soluble Cd in both spikes and spike nodes, increasing by 161.45% and 95.12% compared with CK (Figure 6). Furthermore, it significantly enhanced this ratio in flag leaves and old organizations when compared with CK, increasing by 146.59% and 153.45%, respectively.

### 3.4. Correlation Analysis

**Figure 7:**
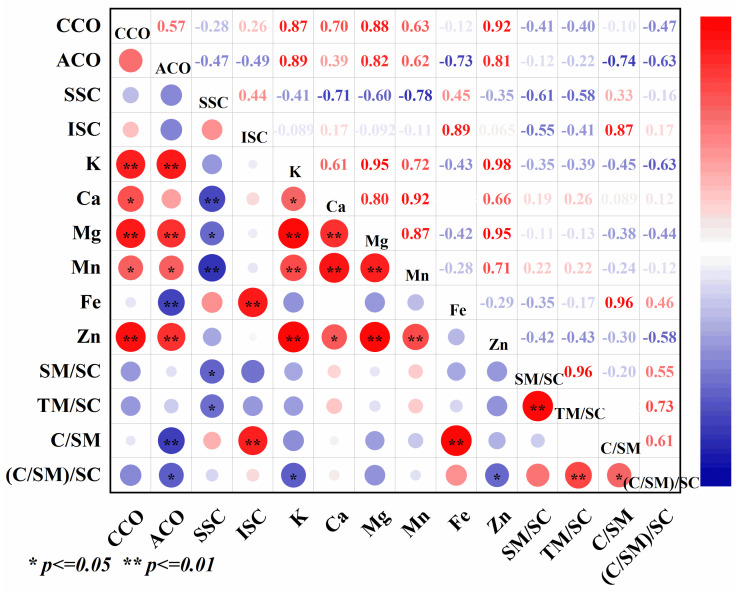
Correlation analysis among the treatments. Data represent the mean of three replicates ± standard deviation. “CCO” indicates Cd content in organs, “ACO” indicates CA content in organs, “SSC” indicates soluble Cd content, “ISC” indicates insoluble Cd content, “SM/SC” indicates soluble Mn/soluble Cd, “TM/SC” indicates total Mn/soluble Cd, “C/SM” indicates Ca/soluble Mn, “(C/SM)/SC” indicates (Ca/soluble Mn)/soluble Cd.

CA could affect the accumulation of Cd in different organs of rice and had a good effect on Cd reduction, and the content of Cd in different organs of rice had a significant effect on the content of mineral elements (Figure 7). Correlation analyses showed that soluble Cd was negatively correlated with the contents of Ca and Mn in different organs of rice, and the differences reached highly significant levels. Soluble Cd was significantly and negatively correlated with Mg and Mn:Cd in different organs of rice, and insoluble Cd was significantly and positively correlated with Fe content and (Ca:Mn). CA content in grains, spikes, spike nodes, flag leaves, inverted second internodes, and roots were positively correlated with Zn, Mn, Mg, and K content, and significantly negatively correlated with Fe and (Ca:Mn).

## 4. Discussion

### 4.1. Foliar Application of CA Could Reduce Rice Absorption and Transport of Cd

Pairs of hydroxyl and carboxyl/carboxyl and carboxyl groups attached to neighboring carbon atoms in CA can form stable five-membered or six-membered cyclic structures by chelating heavy metals within the plant [38]. Under stress conditions, plants may sequester heavy metals in vacuoles, where most heavy metals could be chelated with compounds like CA, thus reducing toxicity risks [39,40]. Research has shown that the addition of organic acid supplements could decrease Cd content in vacuoles, and, through enhancing the isolation of Cd by root vacuoles, thus contribute to maintaining plant growth and reducing Cd content [17]. The foliar application of inhibitors could reduce the transport of Cd from rice roots and stem bases to other parts by altering grain quality and lowering rice Cd content [41]. Additionally, when faced with heavy metal stress, CA may protect plant cell-structure integrity and stability by restoring ion balance within the plant. It enhanced cell membrane selectivity for heavy metal ions and formed stable ligand complexes that prevented metal absorption by roots, thus avoiding metal accumulation at sensitive sites in the roots [8,42]. Due to its ability to chelate heavy metals and promote plant growth, bean genotypes with higher secretion levels of CA exhibited lower aluminum (Al) content per unit in their grains [43]. Exposed to heavy metal stress, the foliar application of thiol compounds could inhibit the migration of Cd, Pb, and As within rice flag leaves, thereby reducing the accumulation of relevant heavy metals in grains [44]. This suggests that foliar spraying methods may enhance the metal-blocking capacity of specific rice organs, leading to a decrease in Cd content in rice grains.

Our research findings indicated that applying CA during the rice flowering stage increased CA content in rice organs such as spikes, spike nodes, flag leaves, inverted nodes, inverted second leaves, and roots. The effect was even better with 5 mM CA compared with 1 mM CA. CA could also reduce Cd content in tissues other than roots and old organizations. Notably, the content of CA in these tissues was significantly negatively correlated with Cd content. Specifically, 5 mM CA significantly reduced Cd content in grains and spikes by 52% and 37%, respectively. This suggests that the foliar application of CA may achieve Cd toxicity reduction by increasing the levels of CA in different rice organs, altering the chemical form of Cd, and chelating and sequestering Cd within these organs, thereby limiting Cd migration to rice grains

Rice organs could convert most of the Cd that entered the plant into insoluble forms, thereby preventing excessive Cd transport to the grains. This metal-blocking effect reached its limit when Cd stress concentrations were high [20]. Research has shown that external applications of CA could convert more Cd in radish stems and leaves into insoluble forms [45]. Our study found that the foliar application of 5 mM CA significantly reduced the soluble Cd content in nutrient-rich organs, other than inverted two leaves and old organizations, during the rice flowering stage. Additionally, it markedly decreased the proportion of soluble Cd in spikes, necks of spikes, spike nodes, and roots, while increasing the proportion of insoluble Cd in these organs and then lowering the transfer factor for Cd transport from flag leaves to spikes and from inverted second leaves to inverted nodes. This indicates that the foliar application of CA could enhance the metal-blocking capacity of rice nutrient-rich organs during the rice flowering stage and reduce the transport ability of Cd within the rice plant.

### 4.2. Foliar Application of CA Could Alleviate Cd Content by Increasing the Uptake of Mineral Elements and Reducing the Content of Soluble Cd in Different Organs of Rice

Different elemental ions exhibit synergistic or antagonistic effects. During the growth and development stage of rice, the plant regulates the absorption of essential elements to enhance its ability to withstand external disturbances and maintain normal growth [46]. Under heavy metal stress, the capacity of rice to absorb most essential elements from the roots would be weakened. To sustain normal growth, rice adjusts the levels of elements such as Ca, K, Mg, and Mn to maintain proper antioxidant levels [47]. For instance, when exposed to Cd stress, *Arabidopsis* modulates Ca levels to maintain normal antioxidant systems, with Ca acting as a secondary messenger [48]. Research has identified the FBP (fructose bisphosphatase) gene’s role in mediated antagonistic interactions between certain metal ions within plants. When Fe levels are low and Zn levels are high, FBP expression decreases while NAS (nicotianamine synthase) expression increases, which chelates Fe and Zn and then increases its plant availability. Simultaneously, it reduces Zn transport to the aerial parts, preventing Zn toxicity [49]. In this study, we found that Cd content in various organs of rice was significantly positively correlated with K, Ca, Mg, Mn, and Zn, and Cd content in various organs was significantly reduced under CA treatment, indicating that the application of CA reduced the absorption capacity of Cd in rice and improved the absorption of other mineral elements to maintain the normal growth.

Transport proteins in different nodes of rice mediate the distribution of mineral elements within grains, thereby regulating nutrient uptake, translocation, and distribution [50]. *OsNramp3* (natural resistance-associated macrophage protein) is closely associated with the transport of Mn in rice, and the expression of this transporter-related gene in the xylem and cortex promotes the preferential transfer of Mn to parts such as flag leaves. *OsLCT1* (low-affinity cation transporter) may participate in the relocalization of other elements within leaves, influencing the transport of Cd to rice grains [50,51]. These mechanisms may contribute to the variations in nutrient elements observed in different rice organs under Cd stress.

Acting as a metabolite signal, CA has been shown to play a crucial role in regulating various mineral elements [52,53,54]. Increased CA concentration in soil enhances the ability of plants to cope with Fe deficiency [55]. Not only does CA provide additional anions to cells but it also competes with intracellular phosphate ions for the adsorption of elements like Mn, thereby enhancing their plant availability. At the same time, the binding of phosphate ions with Cd is strengthened, further inhibiting the activity of Cd [56,57]. Our study reveals that, during the rice flowering stage, the foliar application of 5 mM CA significantly increases soluble and insoluble Mn content in grains, spike nodes, and roots; increased Mn content significantly reduces soluble Cd content in rice seeds, spikes, spike nodes, flag leaves, inverted second internodes, and roots, thus reducing Cd transport to various organs and alleviating Cd toxicity.

### 4.3. Foliar Application of CA Alleviates Rice Organ Cd Absorption by Modulating Ca:Mn Ratios

Clarifying the interactions between elements with plant tissues may be an effective solution to mitigate the toxicity of Cd in rice. Currently, the presence of Ca and Mn has been found to reduce the transfer capacity of Cd from roots to shoots and from shoots to grains in cropland soil, thereby mitigating rice grain Cd contamination. Additionally, Cd content is typically positively correlated with Mn content in rice organs [58,59]. Knocking out *OsNramp5* simultaneously reduces Mn and Cd content in different rice organs [60]. Under Cd stress conditions, nutrient-rich organs could alleviate Cd toxicity by adjusting the Ca: Mn ratios. High ratios of Ca: Mn are associated with low Cd accumulation in these organs [20]. Our study reveals that the foliar application of 5 mM CA significantly increases the Mn and soluble Cd ratios in spikes, spike nodes, flag leaves, and roots during the rice flowering stage. This adjustment improves the physiological process of simultaneously absorbing Mn and Cd in rice. Furthermore, it significantly enhances the (Ca:Mn) ratio in spikes, spike nodes, flag leaves, old organizations, and roots in terms of soluble Cd content. At the same time, the increase in (Ca:Mn) would increase the content of insoluble Cd in various organs of rice, reducing the transfer of Cd to rice seeds, and effectively inhibiting rice Cd accumulation.

## 5. Conclusions

During the flowering stage of rice, spraying CA could increase the content of CA in rice organs and reduce the Cd content in tissues other than the root system and old organizations. Compared with 1 mM CA, 5 mM CA made Cd content in grains and spikes significantly decrease by 52% and 37%, respectively. Additionally, 5 mM CA significantly reduced the soluble Cd content in nutrient organs other than the inverted second leaves and old organizations, and in spikes, necks of spikes, spike nodes, and roots. This reduction in soluble Cd allocation was accompanied by an increase in the allocation of insoluble Cd in these organs. Furthermore, the transfer factor for Cd transport from flag leaves to spike nodes and from the inverted second leaves to the inverted nodes decreased, indicating that the inhibitory effect of rice vegetative organs on Cd migration and transport in rice increased. An amount of 5 mM CA also significantly increased the content of Mn in grains, spike nodes, and roots by 21%, 24%, and 33%, respectively. It led to a substantial increase in soluble Mn content in spike nodes and roots by 42% and 20%, respectively, while significantly reducing the content of soluble Cd in rice grain, spikes, spike nodes, flag leaves, inverted second internodes, and roots. Moreover, the ratios of Mn to soluble Cd in spikes, spike nodes, flag leaves, and roots improved the antagonistic effect of Mn and Cd by rice during physiological processes. And the ratio of (Ca:Mn) to soluble Cd in the spikes, spike knots, flag leaves, old organizations, and roots significantly increased. In summary, the foliar application of CA on farmland could decrease the content of soluble Cd, increase the Mn content and the ratios of Ca to Mn of different vegetative organs of rice, and increase the ability of vegetative organs to convert Cd into insoluble Cd, thus reducing the transfer of Cd to the rice grain.

## Figures and Tables

**Table 1:** Correlation between Cd and CA content in rice organisms.

Organ of Rice	Grain	Spike	Neck of Spike	Spike Knots	Flag Leaf	Inverted Two Internodes	Inverted Node	Inverted Two Leaves	Others	Root
Grains		0.90 **	——	0.94 **	0.96 **	——	0.85 **	0.84 **	——	0.93 **
Spikes	0.89 **		——	0.93 **	0.89 **	——	0.80 *	0.81 **	——	0.88 **
Neck of spikes	0.84 **	0.79 *		——	——	——	——	——	0.88 **	——
Spike nodes	0.97 **	0.88 **	0.90 **		0.95 **	——	0.80 **	0.82 **	——	0.92 **
Flag leaves	0.77 *	0.67 *	0.92 **	0.85 **		——	0.84 **	0.75 *	——	0.88 **
Inverted second internodes	0.98 **	0.85 **	0.87 **	0.99 **	0.81 **		——	——	——	——
Inverted nodes	0.95 **	0.77 *	0.78 *	0.93 **	0.67 *	0.96 **		——	——	0.88 **
Inverted second leaves	0.93 **	0.88 **	0.95 **	0.96 **	0.91 **	0.95 **	0.84 **		——	0.79 *
Others	0.78 *	——	0.71 *	0.85 **	0.58	0.87 **	0.89 **	0.76 *		——
Roots	——	0.70 *	——	0.72 *	0.77 *	0.71 *	——	0.81 **	——	
Cd/CA in the same organ	−0.92	−0.89	——	−0.94	−0.81	——	−0.87	−0.86	——	−0.77

Note: Correlation between Cd and CA content in rice organisms is presented on the lower left and the upper right, respectively. ** indicates extremely significant level (*p* < 0.01), * indicates significant level (*p* < 0.05). —— indicates extremely little or no correlation.

**Table 2:** Effects of citric acid on transfer factor (TF) of soluble Cd in organs of rice.

	TF _Grain/Spike_	TF _Spike/SK_	TF _SK/FL_	TF _ITI/IN_	TF _IN/ITL_	TF _ITL/OO_	TF _OO/Roots_
CK	1.3 ± 0.1 a	0.4 ± 0.1 a	141.1 ± 17.7 a	2.5 ± 0.3 a	245.2 ± 44.2 a	8.7 ± 2.3 a	5.0 ± 0.3 a
S1	1.4 ± 0.1 a	0.5 ± 0.1 a	157.7 ± 36.4 a	3.0 ± 0.9 a	155.7 ± 33.3 b	6.5 ± 1.5 a	6.8 ± 2.9 a
S2	1.4 ± 0.3 a	0.6 ± 0.1 a	100.5 ± 13.2 b	2.7 ± 0.7 a	151.9 ± 20.6 b	5.6 ± 1.9 a	6.3 ± 2.0 a

Note: TFA/B = total Cd content in A/soluble Cd content in B. Data represent the means of three repetitions ± standard deviation; lowercase (letters) represent significant differences between different CA treatments (*p* < 0.05). “SK” indicates spike nodes, “FL” indicates flag leaves, “ITI” indicates invert second internodes, “IN” indicates inverted nodes, “ITL” indicates inverted second leaves, “OO” indicates old organizations.

**Table 3:** Effects of CA on essential mineral elements in tissues of rice.

Organ	Treatment	K (g·kg^−1^)	Ca (g·kg^−1^)	Mg (g·kg^−1^)	Mn (g·kg^−1^)	Fe (g·kg^−1^)	Zn (g·kg^−1^)
Grains	CK	0.87 ± 0.03 b	0.57 ± 0.05 a	0.32 ± 0.01 c	0.02 ± 0 b	0.02 ± 0 a	0.02 ± 0 a
	S1	1.04 ± 0.21 b	0.45 ± 0.02 b	0.48 ± 0.05 b	0.02 ± 0 ab	0.02 ± 0 a	0.02 ± 0 b
	S2	1.27 ± 0.05 a	0.44 ± 0.02 b	0.57 ± 0.03 a	0.02 ± 0 a	0.02 ± 0 a	0.02 ± 0 b
Spikes	CK	21.08 ± 1.29 b	1.74 ± 0.05 a	1 ± 0.07 a	0.38 ± 0.03 a	0.13 ± 0.02 b	0.06 ± 0.01 a
	S1	26.44 ± 0.8 a	1.85 ± 0.37 a	1.06 ± 0.11 a	0.35 ± 0.05 a	0.27 ± 0.02 a	0.07 ± 0.01 a
	S2	24.761.05 a	2.12 ± 0.22 a	1.05 ± 0.08 a	0.38 ± 0.03 a	0.36 ± 0.07 a	0.08 ± 0.01 a
Spike nodes	CK	133.92 ± 15.42 a	7.92 ± 0.43 a	5.85 ± 0.89 a	1.42 ± 0.06 b	1.24 ± 0.09 a	1.37 ± 0.06 a
	S1	139.2 ± 26.8 a	7.56 ± 0.24 a	5.42 ± 0.22 a	1.62 ± 0.11 ab	1.44 ± 0.12 a	1.19 ± 0.1 b
	S2	128.76 ± 13.93 a	7.03 ± 0.64 a	4.99 ± 0.49 a	1.76 ± 0.20 a	1.48 ± 0.25 a	1.14 ± 0.01 b
Flag leaves	CK	19.32 ± 1.82 a	5.64 ± 0.26 a	2.38 ± 0.41 a	1.26 ± 0.18 a	0.29 ± 0.03 a	0.05 ± 0.01 a
	S1	21.49 ± 2.68 a	6.17 ± 0.88 a	2.46 ± 0.37 a	1.27 ± 0.08 a	0.33 ± 0.05 a	0.05 ± 0.01 a
	S2	20.28 ± 2.27 a	6.48 ± 0.71 a	2.41 ± 0.45 a	1.14 ± 0.1 a	0.4 ± 0.06 a	0.06 ± 0.01 a
Others	CK	33.47 ± 0.63 a	6.91 ± 0.8 a	2.51 ± 0.2 a	1.13 ± 0.08 a	0.67 ± 0.05 a	0.1 ± 0.01 a
	S1	34.02 ± 0.16 a	7.19 ± 0.15 a	2.44 ± 0.03 a	1.15 ± 0.04 a	0.84 ± 0.07 a	0.09 ± 0.01 a
	S2	31.76 ± 4.82 a	7.15 ± 1.13 a	2.44 ± 0.16 a	0.93 ± 0.1 b	0.87 ± 0.16 a	0.08 ± 0.01 a
Roots	CK	6.55 ± 1.11 a	4.78 ± 0.73 a	1.15 ± 0.19 a	0.66 ± 0.12 b	75.78 ± 6.55 a	0.1 ± 0.02 a
	S1	7.61 ± 1.7 a	4.67 ± 0.07 a	1.19 ± 0.02 a	0.51 ± 0.07 b	67.09 ± 12.85 a	0.09 ± 0.01 a
	S2	5.86 ± 1.4 a	5.65 ± 0.81 a	1.24 ± 0.21 a	0.99 ± 0.06 a	81.42 ± 13.11 a	0.11 ± 0.02 a

Note: Different letters indicate significant difference among treatments in the same organ and same element according to ANOVA analysis of variance (*p* < 0.05). Data represent the means of three repetitions ± standard deviation.

## Data Availability

Data set available on request from the authors.
